# Extended-Interval Dosing Strategy of Immune Checkpoint Inhibitors in Lung Cancer: Will it Outlast the COVID-19 Pandemic?

**DOI:** 10.3389/fonc.2020.01193

**Published:** 2020-06-23

**Authors:** Kartik Sehgal, Daniel B. Costa, Deepa Rangachari

**Affiliations:** ^1^Division of Medical Oncology, Department of Medicine, Beth Israel Deaconess Medical Center and Harvard Medical School, Boston, MA, United States; ^2^Department of Medical Oncology, Dana-Farber Cancer Institute, Boston, MA, United States

**Keywords:** lung cancer, extended-interval dosage, immune checkpoint inhibitors, pembrolizumab, nivolumab, atezolizumab, durvalumab, COVID-19

## Abstract

Patients with lung cancer are particularly vulnerable to complications from coronavirus disease-2019 (COVID-19). Recurrent hospital visits and hospital admission are potential risk factors for acquiring infection with its causative pathogen, severe acute respiratory syndrome coronavirus−2 (SARS-CoV-2). As immune checkpoint inhibitors (ICIs) constitute the therapeutic backbone for the vast majority of patients with advanced lung cancer in the absence of actionable driver oncogenes, there have been intense discussions within the oncology community regarding risk-benefit of delaying these treatments or use of alternative extended-interval treatment strategies to minimize the risk of viral transmission secondary to unintended nosocomial exposures. In the midst of the COVID-19 pandemic, the U.S. Food and Drug Administration (FDA) granted accelerated approval for extended-interval strategy of pembrolizumab at a dose of 400 mg every 6 weeks for all already approved oncologic indications. Herein, we summarize the evidence from the *in silico* pharmacokinetic modeling/simulation studies supporting extended-interval dosing strategies for the ICIs used in lung cancer. We further review the evolving clinical evidence behind these approaches and predict that they will continue to be used in routine practice even long after the pandemic, particularly for patients with durable disease control.

## Introduction

Immune checkpoint inhibitors (ICIs) have acquired an indisputable place in the evidence-based care of lung cancers (1). Drugs targeting the programmed death (PD)-1/PD-ligand-1 (PD-L1) and cytotoxic T lymphocyte antigen (CTLA)-4 pathways have been approved, either as monotherapy or in combination with other agents, for management of locally advanced and metastatic non-small cell lung cancer (NSCLC) by the United States (U.S.) Food and Drug Administration (FDA) and other regulatory agencies around the world (Table 1). These include PD-1 inhibitors (pembrolizumab and nivolumab), PD-L1 inhibitors (atezolizumab and durvalumab), and a CTLA-4 inhibitor (ipilimumab). Additionally, PD-L1 inhibitors have been approved in combination with chemotherapy by the U.S. FDA for first line management of extensive-stage small cell lung cancer (SCLC), while PD-1 inhibitors are currently approved in third or later-line treatment settings for metastatic SCLC (Table 1).

**Table 1:** Dosing strategies of immune checkpoint inhibitors approved for lung cancer by the U.S. Food and Drug Administration.

		**Anti-PD-1 antibodies**		**Anti-PD-L1 antibodies**		**Anti-CTLA4 antibody**
		**Pembrolizumab**	**Nivolumab**	**Atezolizumab**	**Durvalumab**	**Ipilimumab**
Metastatic or advanced unresectable NSCLC (First or later-line settings)	Single agent (either monotherapy or maintenance therapy)	200 mg every 3 weeks **OR** 400 mg every 6 weeks	240 mg every 2 weeks **OR** 480 mg every 4 weeks	1200 mg every 3 weeks **OR** 840 mg every 2 weeks **OR** 1680 mg every 4 weeks		
	Along with chemotherapy	200 mg every 3 weeks **OR** 400 mg every 6 weeks	240 mg every 2 weeks **OR** 480 mg every 4 weeks	1200 mg every 3 weeks		
	Along with chemotherapy + bevacizumab			1200 mg every 3 weeks		
	Combination immunotherapy		3 mg/kg every 2 weeks (+ Ipilimumab 1 mg/kg every 6 weeks)			1 mg/kg every 6 weeks (+ Nivolumab 3 mg/kg every 2 weeks)
	Combination immunotherapy along with 2 cycles of chemotherapy		360 mg every 3 weeks (+ Ipilimumab 1 mg/kg every 6 weeks)			1 mg/kg every 6 weeks (+ Nivolumab 360 mg every 3 weeks)
Unresectable Stage III NSCLC (after concurrent chemoradiation therapy)	Single agent as maintenance therapy				10 mg/kg every 2 weeks	
Extensive stage SCLC	Along with chemotherapy			1200 mg every 3 weeks	1500 mg every 3 weeks	
	Single agent as maintenance therapy			1200 mg every 3 weeks **OR** 840 mg every 2 weeks **OR** 1680 mg every 4 weeks	1500 mg every 4 weeks	
Extensive stage/recurrent metastatic SCLC (progression on/after platinum-based chemotherapy and at least one other line of therapy)	Single agent	200 mg every 3 weeks **OR** 400 mg every 6 weeks	240 mg every 2 weeks			

*CTLA-4, Cytotoxic T lymphocyte antigen-4; NSCLC, Non-small cell lung cancer; PD-1, Programmed death-1; PD-L1, Programmed death ligand-1; SCLC, Small cell lung cancer*.

Coronavirus disease-2019 (COVID-19) is an ongoing global pandemic which has caused more than 380,000 deaths worldwide as of June 4th, 2020 (2). Patients with cancer are among the most vulnerable groups for infection with its causative pathogen, severe acute respiratory syndrome coronavirus-2 (SARS-CoV-2), and can develop severe morbidity and mortality from COVID-19 (3–8). Even among all cancer patients, those with lung cancer appear to be at a disproportionately higher risk for poor outcomes (3, 7–10). In a preliminary analysis of 200 patients with lung cancer and COVID-19 enrolled in the global TERAVOLT (Thoracic cancERs international coVid 19 cOLlaboratTion) registry, the mortality rate was 33.3% (10). This increased risk may be attributable to association of lung cancer with advanced age, smoking, preexisting lung damage and therapy-related immune impairment– all risk factors for severe COVID-19 complications (11). The specific impact of active treatment with ICIs in patients with cancer who develop COVID-19 is not entirely clear (12). While ICIs may theoretically promote immunocompetence to fight viral infection, they may also potentiate hyperinflammation associated with severe COVID-19. Anecdotal case reports of fatalities have been described in patients with advanced lung cancer who were receiving treatment with ICIs (13). However, a single-center study assessing 69 consecutive patients with lung cancer and COVID-19 did not find an association between PD-1 blockade and severity of COVID-19 after adjustment for smoking status (9).

Hospital admissions and recurrent hospital visits have been identified as potential risk factors for infection with SARS-CoV-2 (3, 5). To minimize the risk of hospital-acquired transmission, there have been fervent discussions within the oncology community about the risk-benefit of delaying/extending intervals of treatment cycles for those receiving ICIs as part of routine care (12). The majority of ICIs were initially approved as either every 2 weeks (Q2W) or every 3 weeks (Q3W) dosing regimens. Extended-interval dosing of nivolumab and atezolizumab have subsequently been approved based on *in silico* (modeling/simulation) studies (14, 15). The extended-interval frequency of treatment with pembrolizumab (the most commonly utilized ICI in the first-line setting for advanced NSCLC) at 400 mg every 6 weeks (Q6W) was recently granted accelerated approval by the U.S. FDA on April 28, 2020, thereby providing an evidence-based option for less frequent treatment of patients with lung and other cancers for which pembrolizumab has previously obtained approval. Herein, we summarize the pharmacokinetic/pharmacodynamic and clinical evidence behind extended-interval dosing regimens of ICIs currently approved by the U.S. FDA for management of patients with lung cancer.

## Lessons Learnt From Early Phase Development Trials

Early drug development studies provided data on pharmacokinetic (PK) and pharmacodynamic (PD) properties of immune-checkpoint inhibitors, including anti-PD-1, anti-PD-L1 and anti-CTLA-4 antibodies (1, 16, 17). These are either humanized or fully human monoclonal immunoglobulin (IgG)-1 antibodies, with the exception of the PD-1 inhibitors pembrolizumab and nivolumab, which are IgG4 molecules. As with other monoclonal antibodies, they exhibit a low volume of distribution, low clearance, and long half-lives; their clearance is also minimally affected by renal or hepatic impairment (16, 17).

Two compartment models best characterize the PK properties of ICIs (16). Over the dose range studied, linear PK profiles with time-varying clearance have been described for: pembrolizumab (0.3–10 mg/kg) (18–20), nivolumab (0.1–20 mg/kg) (21–23), atezolizumab [1–20 mg/kg including 1,200 mg dose) (24), and durvalumab (>3 mg/kg) (25, 26). A time-invariant linear PK model characterizes ipilimumab (0.3–10 mg/kg) (27, 28). However, at lower dose ranges, the PK profile has been found to be non-linear for pembrolizumab (<0.3 mg/kg) (18, 19) and durvalumab (<3 mg/kg) (25, 26). Steady-state exposure is achieved after treatment for ~19 weeks with pembrolizumab (Q3W) (29), 12 weeks with nivolumab (Q2W) (21, 22), and 16 weeks with durvalumab (Q2W) (26). In the dose-escalation studies, maximal tolerated dose was not reached for pembrolizumab (0.005-10 mg/kg) (19), nivolumab (0.1–10 mg/kg) (21, 30, 31), atezolizumab (0.01–20 mg/kg) (32–34), or durvalumab (0.1–15 mg/kg) (35). PD analyses have shown maximal occupancy of PD-1 receptors with nivolumab at doses as low as 0.1–0.3 mg/kg (21, 30) and with duvalumab at ≥0.3 mg/kg (36), while maximal effect on lymphocyte stimulation was seen with pembrolizumab at doses ≥1 mg/kg (19).

Clinicopathological features are known to influence the PKs of monoclonal antibodies and may contribute to interpatient variability (16, 37). Body weight is the most important patient variable influencing clearance of ICIs. Other patient characteristics such as age, sex, ethnicity, performance status, tumor type, initial tumor burden, serum albumin level, initial lactate dehydrogenase level, liver function, and renal function have not been shown to affect PK parameters in a clinically relevant manner (20, 22, 25, 27, 29). An exception is a prospective study of nivolumab in NSCLC, melanoma and renal cancer, where sex and baseline serum albumin significantly affected drug clearance in addition to body surface area (38). Finally, development of anti-drug antibodies (ADA) has not been associated with a clinically significant deleterious effect on PKs or efficacy of pembrolizumab (39), nivolumab (40), atezolizumab (24), or durvalumab (26). However, the effect of ADA on drug clearance merits further investigation, as a negative association with overall survival has been found for ipilimumab in patients with advanced melanoma (41), and conflicting results have been reported for nivolumab in the post-drug approval setting (16).

## Exposure-Matching: Leveraging Population Pharmacokinetic Modeling

Population pharmacokinetic (PPK) modeling and simulation studies have relied on data collected in the early dose-ranging studies of ICIs. Flat exposure-response relationship with a wide therapeutic index has been described for pembrolizumab in patients with NSCLC (42, 43) and other solid malignancies (19, 44–46). Similar results were seen in a study which retrospectively pooled data from KEYNOTE-002 (melanoma) and KEYNOTE-010 (NSCLC), with subsequent prospective validation in data from KEYNOTE-024 (NSCLC) (47). In addition, drug clearance has also been associated with patient outcomes (survival/response) with pembrolizumab treatment (47, 48). With regards to nivolumab, exposure concentrations reached plateau at doses ≥3 mg/kg for patients with NSCLC and at ≥1 mg/kg for patients with melanoma in a phase I dose-escalation study (21, 22, 30). Most subsequent studies have reported relatively flat dose/exposure-response for nivolumab with a wide therapeutic index in patients with NSCLC (49, 50) and other solid malignancies (51–53). However, a real-world study in 76 patients with metastatic NSCLC reported a potential exposure-response relationship with nivolumab treatment at the 3 mg/kg dose (54). Higher trough concentrations were seen in patients with longer overall survival; however, this was not adjusted for clinical or biological factors. Multiple studies have consistently demonstrated baseline clearance to be a strong predictor of objective response and overall survival on treatment with nivolumab across multiple tumor types (30, 51, 52), including NSCLC (38, 49). In addition, decrease in clearance over time has also been found as an independent predictor of objective response (23). Atezolizumab has also been shown to have a flat exposure-response profile (including 1200 mg Q3W dosing) with regards to efficacy and safety in urothelial carcinoma (24). In contrast, exposure-response relationship for efficacy and safety has been consistently reported with ipilimumab treatment in patients with advanced melanoma, supporting its body weight-based dosing (55–57).

Fixed flat dosing of PD-1/PD-L1 ICIs has been evaluated using PPK and exposure-response analyses in modeling/simulation studies. This is consistent with the model-informed drug development approach endorsed by the U.S. FDA, where *in silico* studies may provide primary evidence for alternative dosing strategies when effectiveness is well-established in other settings (58, 59). These *in silico* studies with PD-1/PD-L1 inhibitors predicted comparable exposures between flat and traditional body weight-based dosing strategies (25, 60, 61), leading to regulatory approval of fixed dose regimens for nivolumab and pembrolizumab. Interestingly, the 1200 mg Q3W flat dose of atezolizumab was selected early during drug development process, as it achieved preclinically determined target serum tough concentration of 6 μg/mL in >95% of patients (24, 62).

A potential limitation of *in silico* studies is the inability to fully account for drug clearance, development of ADA and other clinicopathological variables. The simulated exposure concentrations require prospective validation in biologically dynamic systems. In addition, the vast majority of these studies have either been performed or provided funding by manufacturing pharmaceutical companies, which may have been a factor in the selection of the dose and frequency chosen for comparisons (63).

## Exposure-Matching: Development of Extended-Interval Dosing Strategies

The model-based approach was further expanded to evaluate extended-interval frequencies based on the aforementioned principles and data. The initial study with nivolumab compared the standard 3 mg/kg Q2W and 240 mg Q2W regimens with the extended-interval strategy of 480 mg every 4 weeks (Q4W) (14, 58). This study utilized steady-state PK measures of trough concentration (C_min_) and time-averaged concentration as surrogate markers for efficacy, while peak concentration (C_max_) was used as a surrogate marker for safety. Using data from 3,817 patients from clinical trials across tumor types (including NSCLC and SCLC), nivolumab administration at 480 mg Q4W was predicted to lead to similar steady-state time-averaged concentrations, 16% lower C_min_ and 45% higher C_max_ compared to traditional 3 mg/kg Q2W dosing. Notably, a modest increase in interpatient variability in exposure was detected with the extended-interval regimen. The predicted C_max_ was lower than that of 10 mg/kg Q2W, the maximal dose evaluated and well-tolerated in the dose-escalation trials. Another simulation study predicted comparable clinical outcomes (objective response rate, overall survival) for patients with NSCLC, melanoma, and renal cell cancer with nivolumab 480 mg Q4W compared to 3 mg/kg Q2W regimen (64). These data collectively led to the approval of nivolumab 480 mg Q4W in patients with previously treated advanced NSCLC.

Subsequently, another modeling study simulated pembrolizumab concentration time profiles by utilizing the PPK model based on data from 2,993 participants in five clinical trials of patients with multiple tumor types (65). This study also measured steady-state PK profiles to compare efficacy and safety of pembrolizumab 400 mg Q6W with the standard doses of 200 mg Q3W and 2 mg/kg Q3W. Of the two efficacy parameters assessed, area under the curve (AUC) was nearly identical between the different groups. The more conservative estimate of mean C_min_ for 400 mg Q6W regimen was predicted to be 12% and 34% lower compared to 200 mg Q3W and 2 mg/kg Q3W dosing, respectively. Only ~0.5% patients with the 400 mg Q6W dose had C_min_ below the lowest value for 2 mg/kg Q3W for an average of 3 days. This was thought to not have a significant effect on target saturation of PD-1 receptors, as it was predicted that reduced concentrations would be required for at least 7 days (based on five half-lives of receptor turnover) for replacement of steady state levels of PD-1. The surrogate measure for safety- C_max_ with 400 mg Q6W regimen was predicted to be lower at all times when compared to 10 mg/kg Q2W, the highest/most frequent pembrolizumab dose administered in clinical trials. The authors further demonstrated that these PK profiles were consistent across tumor types with a flat exposure-response relationship over a five-fold dose range. These data ultimately led to approval of pembrolizumab 400 mg Q6W for all previously approved indications by the U.S. FDA on April 28, 2020.

A similar exposure-matching study with atezolizumab employed data from three clinical trials: PCD4989g (NSCLC and urothelial carcinoma cohorts), OAK (NSCLC), and IMvigor211 (urothelial carcinoma) (15). The predicted exposure with extended-interval dosing at 1680 mg Q4W was comparable to the standard 1200 mg Q3W regimen. At steady-state, the predicted serum C_min_ with 1680 mg Q4W was 6% lower than that for 1200 mg Q3W regimen– but remained tenfold higher than the target serum C_min_ of 6 μg/mL. The predicted AUC was 4.8% higher than that for 1200 mg Q3W. Further evaluation revealed no clear exposure-safety relationship for atezolizumab. The predicted C_max_ with 1680 mg Q4W was within the range observed with the maximal 20 mg/kg dose tested in the dose-escalation study PDC4989g, with a comparable safety profile demonstrated in those below or above this predicted C_max_. This study contributed to the US. FDA approval of the 1680 mg Q4W atezolizumab regimen when used as monotherapy in first or later-line settings.

## Clinical Evidence Behind Extended-Interval Dosing Strategies

The regulatory approval of extended-interval strategies of ICIs has been predominantly based on the aforementioned *in silico* studies. While these strategies have the potential to improve patient experience and reduce infusion center-related costs, concerns remain regarding less frequent monitoring for progression and immune-related adverse events with potentially serious consequences. The clinical evidence behind use of these strategies is still evolving.

The safety profile of the nivolumab 480 mg Q4W regimen was evaluated in a pooled dataset of 61 patients from four phase III (CheckMate 066, 025, 057, and 017) clinical trials (14). This study included patients who either transitioned to the extended-interval nivolumab strategy or crossed over after disease progression in the comparator arm in the open-label phase. When compared to data available for 1,030 patients receiving the 3 mg/kg Q2W regimen, the incidence of treatment-related adverse events (TRAEs, 14.8%) was similar. Ongoing randomized clinical trials evaluating the nivolumab 480 mg Q4W schedule include phase IIIB/IV CheckMate 384 (NCT02713867) in previously treated advanced NSCLC and CheckMate 511 (NCT02714218) in advanced melanoma. CheckMate 384 is an international open-label randomized study comparing 480 mg Q4W and 240 mg Q2W regimens in patients with previously treated stage IIIB/IV or recurrent NSCLC who have received prior treatment with nivolumab 240 mg Q2W for ≤12 months with ≥2 consecutive response assessments without evidence of disease progression. In an interim analysis of 329 patients (166 in 480 mg Q4W and 163 in 240 mg Q2W groups) with median follow up of 9.5 months (Q4W) and 10.2 months (Q2W), respectively, endpoints of efficacy and safety were comparable (66). The co-primary endpoint of post-randomization 6-months progression-free survival (PFS) rates for 480 mg Q4W vs. 240 mg Q2W regimens were 75 vs. 80%, respectively; 12-month PFS rates were 53% in both groups. Median PFS rates for 480 mg Q4W vs. 240 mg Q2W regimens were 12.1 vs. 12.2 months, respectively (HR, 0.96). Any grade TRAEs were seen in 48% and 61% of patients on 400 mg Q4W and 200 mg Q2W nivolumab doses, respectively. The other CheckMate 511 study is utilizing 480 mg Q4W nivolumab regimen in the maintenance phase for treatment of advanced melanoma for the two main cohorts (67). In the third cohort of the trial not included in the original protocol, 27 patients have been randomized to initial treatment with 6 mg/kg nivolumab and ipilimumab 1 mg/kg, followed by 480 mg every 8 weeks (Q8W) nivolumab in the maintenance phase. Preliminary results available at ClinicalTrials.gov showed comparable rates of serious adverse events (37.0%), other (not including serious) adverse events (96.3%), and all-cause mortality (25.9%) in this cohort as compared to the other two main cohorts studying different combinations of nivolumab and ipilimumab at 1–3 mg/kg doses. Nivolumab 360 mg Q3W has most recently been approved in combination with ipilimumab and 2 cycles of platinum-doublet chemotherapy for first line treatment of advanced NSCLC based on results from CheckMate 9LA trial (NCT03215706) (68).

The extended-interval 400 mg Q6W regimen of pembrolizumab is currently being evaluated in 100 patients with advanced melanoma in cohort B of the ongoing Phase I open-label KEYNOTE-555 study (NCT03665597). Interim analysis of results from the first 44 patients enrolled in this cohort showed overall response rate (primary endpoint) of 38.6% (95% CI, 24.4-54.5) and complete response rate of 9.1% (69). These are similar to the historically reported response rates with pembrolizumab in metastatic melanoma. Additionally, exposure concentrations observed in patients in this study with the 400 mg Q6W regimen were within the 90% prediction intervals of simulated concentrations from the model-based study (65). C_min_ with 400 mg Q6W was 18% lower than 200 mg Q3W, while C_max_ was 38% lower than the maximum clinically tested dose of 10 mg/kg Q2W. The safety profile of the Q6W regimen was also comparable to that seen with the Q3W regimen in clinical trials, with grade 3-4 all-cause adverse events reported in 25.0% patients. These interim results were included in the resubmitted Supplemental Biologics License Application, which ultimately led to approval of the 400 mg Q6W pembrolizumab regimen by the U.S. FDA.

No completed clinical trials have yet evaluated the extended-interval atezolizumab dosing regimen of 1680 mg Q4W; 1200 mg Q3W was utilized in all landmark IMpower trials in advanced NSCLC and extensive-stage SCLC (15). The 1680 mg Q4W regimen of atezolizumab has, however, been approved based on *in silico* studies for all indications when used as monotherapy. Ongoing clinical trials in lung cancer which have incorporated the 1680 mg Q4W regimen include NCT04267237 (in both study arms) and NCT03178552 (in experimental cohort being compared to chemotherapy).

The phase III CASPIAN trial (NCT03043872), which led to approval of durvalumab in extensive-stage SCLC, evaluated a dose of 1500 mg Q3W in combination with chemotherapy in the initial phase; then followed by 1500 mg Q4W regimen in the maintenance phase (70). In NSCLC, durvalumab is currently approved only at the dose of 10 mg/kg Q2W for maintenance therapy after curative intent chemoradiation therapy for patients with stage IIIB disease.

Ipilimumab is the most recently approved ICI for management of advanced NSCLC in the first-line setting. It has been approved at a dose of 1 mg/kg Q6W in combination with nivolumab 3 mg/kg Q2W, as evaluated for patients with tumor PD-L1 expression in the CheckMate 227 trial (NCT02477826) (71), as well as in combination with nivolumab 360 mg Q3W and 2 cycles of platinum-doublet chemotherapy, as evaluated in the CheckMate 9LA trial (NCT03215706) (68).

## Alternative Dosing Regimens in the Real-World

The wide therapeutic index and flat exposure-response profiles seen with ICIs have raised additional questions regarding strategies that would permit the lowest and least frequent dosage of the drug with optimal efficacy outcomes (72, 73). Few real-world studies have evaluated administration of standard FDA-approved (or lower) doses of ICIs at extended intervals. A multicenter retrospective study from Israel with median follow up of 35.6 months found no significant differences in PFS for patients with previously treated advanced NSCLC who received nivolumab for >2 years at approved doses (3 mg/kg Q2W/ 240 mg Q2W/ 480 mg Q4W) (*N* = 25) vs. extended-interval doses (3 mg/kg Q3W- Q8W) (*N* = 13) (74). Another study from South Korea retrospectively evaluated outcomes of 18 patients with advanced NSCLC treated with low doses of nivolumab chosen to approximate 0.1 mg/kg and 1 mg/kg [20 mg [*N* = 3] or 100 mg [*N* = 15], respectively] Q3W (due to financial toxicity) compared to 29 patients who received standard dose nivolumab (3 mg/kg) Q3W (75). At mean follow up of 5.2 months, the authors reported no significant differences in the response rates, PFS, or overall survival between the two groups. In another retrospective multicenter study, our group evaluated association of treatment delays/extensions with outcomes in patients with advanced NSCLC who received at least four cycles of pembrolizumab treatment. Similar outcomes were seen in those who received two or more pembrolizumab 200 mg Q3W cycles at extended-intervals (>3 weeks + 3 days) due to immune-related adverse events, medical reasons, or patient-physician preferences (*N* = 27), as compared to those who received all (or up to one non-standard) treatment cycles on the standard 200 mg Q3W schedule (*N* = 65) (76). All these studies, however, are limited by the retrospective nature of their analyses and small sample sizes, making these results at best hypothesis generating. In addition, the evolution of disease biology and patient characteristics may affect outcomes in those with extension of the ICI intervals after achieving disease stability/response. This data should not be extrapolated to those at the beginning of ICI therapy.

## Economics of Extended-Interval Dosing Strategies

The rapid adoption of ICIs in routine practice has had a substantial financial impact on global health care systems (73, 77, 78). Cost analyses have been performed to compare different dosing strategies including body-weight based, fixed/flat, PK-derived, and banded dosing (79). While multiple studies have predicted cost-savings with personalized body-weight administration of ICIs compared to fixed/flat dosing (80–82), data is sparse on cost impact of extended-interval dosing strategies. A simulation study based on a PPK model of nivolumab predicted a potential for 70% cost-savings if nivolumab 480 mg was administered every 8 to 14 weeks following two initial doses at 480 mg Q4W (83). Another study proposed therapeutic drug monitoring of exposure concentrations to evaluate potential use of nivolumab 480 mg Q8W or longer in selected patients (72).

## Potential Pitfalls of Extended-Interval Dosing Strategies

The aforementioned clinical evidence supporting extended-interval dosing regimens is still preliminary. The impact on monitoring for clinical progression, especially in patients with SCLC, and detection of immune-related adverse events is yet undefined. This may, however, be potentially addressed by incorporation of telemedicine visits for symptom check-in and laboratory testing with local partners in the community. Another potential pitfall is the applicability of data derived from PPK modeling from patients with multiple types of malignancies to patients with NSCLC and SCLC. Whether patients with different tumor histologies and treatment characteristics would have different benefit-to-risk ratios from extended-interval strategies is not fully understood. Moreover, the consequences of longer intervals on eligibility for subsequent clinical trials, which incorporate timing from the last ICI dose in the inclusion criteria, still need to be addressed. Finally, patient-reported outcomes have not been described yet, making the potential advantage of improved patient experience an assumption at this time.

## Conclusions and Future Directions

Knowledge of ICI PK properties from dose-ranging studies during early drug development has proven fruitful in facilitating subsequent modeling and simulation efforts that have permitted exploration of the efficacy and safety of extended-interval dosing strategies, thus resulting in regulatory approval for multiple ICIs currently used in routine clinical practice. These data clearly highlight the importance of well-designed early phase drug trials incorporating wide dose-ranging strategies. Despite potential limitations of *in silico* studies to fully account for clearance, ADA, and other clinicopathologic variables, predictions of the performance characteristics of extended-interval pembrolizumab have since been clinically validated in the preliminary results of the KEYNOTE-555 trial (69).

As the indications for their use continue to expand, optimization of access to, use of, and patient-experience with ICIs will entail continued exploration of strategies that will permit their application at the lowest dose and longest intervals with maximal efficacy. For any given ICI, this remains yet to be determined. Modeling and simulation studies utilizing PPK models have the potential to answer these questions with nominal costs. Therapeutic drug monitoring of ICIs, while taking into account time-varying clearance of PD-1/PD-L1 inhibitors, is another strategy which has the potential to personalize dosing and result in cost-savings (63, 72). Integration of biomarkers such as circulating tumor-free DNA, T-cell receptor expansion, and inflammatory/nutritional surrogates may help guide the optimal timing for switch from standard to extended-interval regimens. Finally, early incorporation of pharmacoeconomic analyses during the drug development phase may allow selection of the most cost-effective regimen.

Extended-interval dosing strategies have the potential to improve patient-reported outcomes by providing flexibility and convenience to both patients and their caregivers, while reducing hospital and infusion-related costs. This has acquired additional importance during the global COVID-19 pandemic. The availability of more flexible dosing regimens provides an evidence-based approach to minimize healthcare exposure to SARS-CoV-2 for this vulnerable population without compromising therapeutic efficacy. The clinical data supporting continued use of extended-interval strategies in routine settings is, however, still evolving. Outside unprecedented situations such as the COVID-19 pandemic, these will likely be the most attractive for patients with lung cancer who have already achieved disease control with ICIs. Individual patient level considerations, however, are warranted to balance the convenience of these extended-interval regimens with the appropriate interval of patient assessment for clinical progression and immune-related adverse events.

## Author Contributions

KS was responsible for manuscript design. The final article was critically reviewed and approved for publication by KS, DC, and DR. All authors contributed to the writing and editing process and collaborated on the manuscript.

## Conflict of Interest

DR reports non-financial support (institutional research support) from Bristol-Myers Squibb, Novocure, and Abbvie/Stemcentrx, all outside the submitted work. DC reports personal fees (consulting fees and honoraria) and non-financial support (institutional research support) from Takeda/Millennium Pharmaceuticals, AstraZeneca, and Pfizer, as well as non-financial support (institutional research support) from Merck Sharp and Dohme Corporation, Merrimack Pharmaceuticals, Bristol-Myers Squibb, Clovis Oncology, Spectrum Pharmaceuticals and Tesaro, all outside the submitted work. The remaining author declares that the research was conducted in the absence of any commercial or financial relationships that could be construed as a potential conflict of interest.

## References

[1] Remon J Passiglia F Ahn MJ Barlesi F Forde PM Garon EB. Immune checkpoint inhibitors in thoracic malignancies: review of the existing evidence by an IASLC expert panel and recommendations. J Thorac Oncol. (2020) 15:914–47. 10.1016/j.jtho.2020.03.006 32179179

[2] Dong E Du H Gardner L. An interactive web-based dashboard to track COVID-19 in real time. Lancet Infect Dis. (2020) 20:533–4. 10.1016/S1473-3099(20)30120-1 32087114 PMC7159018

[3] Yu J Ouyang W Chua MLK Xie C. SARS-CoV-2 transmission in patients with cancer at a tertiary care hospital in Wuhan, China. JAMA Oncol. (2020) 25:e200980. 10.1001/jamaoncol.2020.0980 32211820 PMC7097836

[4] Liang W Guan W Chen R Wang W Li J Xu K. Cancer patients in SARS-CoV-2 infection: a nationwide analysis in China. Lancet Oncol. (2020) 21:335–7. 10.1016/S1470-2045(20)30096-6 32066541 PMC7159000

[5] Zhang L Zhu F Xie L Wang C Wang J Chen R. Clinical characteristics of COVID-19-infected cancer patients: a retrospective case study in three hospitals within Wuhan, China. Ann Oncol. (2020) 31:894–901. 10.1016/j.annonc.2020.03.296 32224151 PMC7270947

[6] Desai A Sachdeva S Parekh T Desai R. COVID-19 and cancer: lessons from a pooled meta-analysis. JCO Glob Oncol. (2020) 6:557–9. 10.1200/GO.20.00097 32250659 PMC7193801

[7] Dai M Liu D Liu M Zhou F Li G Chen Z. Patients with cancer appear more vulnerable to SARS-COV-2: a multicenter study during the COVID-19 outbreak. Cancer Discov. (2020) 10:783–91. 10.1158/2159-8290.CD-20-0422 32345594 PMC7309152

[8] Mehta V Goel S Kabarriti R Cole D Goldfinger M Acuna-Villaorduna A. Case fatality rate of cancer patients with COVID-19 in a New York Hospital System. Cancer Discov. (2020). 10.1158/2159-8290.CD-20-0516. [Epub ahead of print]. 32357994 PMC7334098

[9] Luo J Rizvi H Egger JV Preeshagul IR Wolchok JD Hellmann MD. Impact of PD-1 blockade on severity of COVID-19 in patients with lung cancers. Cancer Discov. (2020). 10.1158/2159-8290.CD-20-0596. [Epub ahead of print]. 32398243 PMC7416461

[10] Garassino MC editor TERAVOLT (Thoracic cancERs international coVid 19 cOLlaboraTion): First results of a global collaboration to address the impact of COVID-19 in patients with thoracic malignancies. AACR Annual Meeting (2020).

[11] Passaro A Peters S Mok TSK Attili I Mitsudomi T de Marinis F. Testing for COVID-19 in lung cancer patients. Ann Oncol. (2020) 31:832–4. 10.1016/j.annonc.2020.04.002 32278879 PMC7144604

[12] Bersanelli M. Controversies about COVID-19 and anticancer treatment with immune checkpoint inhibitors. Immunotherapy. (2020) 12:269–73. 10.2217/imt-2020-0067 32212881 PMC7117596

[13] Bonomi L Ghilardi L Arnoldi E Tondini CA Bettini AC. A rapid fatal evolution of Coronavirus Disease-19 (COVID-19) in an advanced lung cancer patient with a long time response to nivolumab. J Thorac Oncol. (2020) 15:e83–5. 10.1016/j.jtho.2020.03.021 32243919 PMC7270552

[14] Long GV Tykodi SS Schneider JG Garbe C Gravis G Rashford M. Assessment of nivolumab exposure and clinical safety of 480 mg every 4 weeks flat-dosing schedule in patients with cancer. Ann Oncol. (2018) 29:2208–13. 10.1093/annonc/mdy408 30215677 PMC6290887

[15] Morrissey KM Marchand M Patel H Zhang R Wu B Phyllis Chan H. Alternative dosing regimens for atezolizumab: an example of model-informed drug development in the postmarketing setting. Cancer Chemother Pharmacol. (2019) 84:1257–67. 10.1007/s00280-019-03954-8 31542806 PMC6820606

[16] Desnoyer A Broutin S Delahousse J Maritaz C Blondel L Mir O Pharmacokinetic/pharmacodynamic relationship of therapeutic monoclonal antibodies used in oncology: part 2, immune checkpoint inhibitor antibodies. Eur J Cancer. (2020) 128:119–28. 10.1016/j.ejca.2020.01.003 32037060

[17] Centanni M Moes D Troconiz IF Ciccolini J van Hasselt JGC. Clinical pharmacokinetics and pharmacodynamics of immune checkpoint inhibitors. Clin Pharmacokinet. (2019) 58:835–57. 10.1007/s40262-019-00748-2 30815848 PMC6584248

[18] Elassaiss-Schaap J Rossenu S Lindauer A Kang SP de Greef R Sachs JR. Using model-based “Learn and Confirm” to reveal the pharmacokinetics-pharmacodynamics relationship of pembrolizumab in the KEYNOTE-001 trial. CPT Pharmacometrics Syst Pharmacol. (2017) 6:21–8. 10.1002/psp4.12132 27863143 PMC5270295

[19] Patnaik A Kang SP Rasco D Papadopoulos KP Elassaiss-Schaap J Beeram M. Phase I study of pembrolizumab (MK-3475; anti-PD-1 monoclonal antibody) in patients with advanced solid tumors. Clin Cancer Res. (2015) 21:4286–93. 10.1158/1078-0432.CCR-14-2607 25977344

[20] Ahamadi M Freshwater T Prohn M Li CH de Alwis DP de Greef R. Model-based characterization of the pharmacokinetics of pembrolizumab: a humanized anti-PD-1 monoclonal antibody in advanced solid tumors. CPT Pharmacometrics Syst Pharmacol. (2017) 6:49–57. 10.1002/psp4.12139 27863186 PMC5270291

[21] Topalian SL Hodi FS Brahmer JR Gettinger SN Smith DC McDermott DF. Safety, activity, and immune correlates of anti-PD-1 antibody in cancer. N Engl J Med. (2012) 366:2443–54. 10.1056/NEJMoa1200690 22658127 PMC3544539

[22] Bajaj G Wang X Agrawal S Gupta M Roy A Feng Y. Model-based population pharmacokinetic analysis of nivolumab in patients with solid tumors. CPT Pharmacometrics Syst Pharmacol. (2017) 6:58–66. 10.1002/psp4.12143 28019091 PMC5270302

[23] Liu C Yu J Li H Liu J Xu Y Song P. Association of time-varying clearance of nivolumab with disease dynamics and its implications on exposure response analysis. Clin Pharmacol Ther. (2017) 101:657–66. 10.1002/cpt.656 28182273

[24] Stroh M Winter H Marchand M Claret L Eppler S Ruppel J. Clinical Pharmacokinetics and Pharmacodynamics of Atezolizumab in Metastatic Urothelial Carcinoma. Clin Pharmacol Ther. (2017) 102:305–12. 10.1002/cpt.587 27981577

[25] Baverel PG Dubois VFS Jin CY Zheng Y Song X Jin X. Population Pharmacokinetics of Durvalumab in Cancer Patients and Association With Longitudinal Biomarkers of Disease Status. Clin Pharmacol Ther. (2018) 103:631–42. 10.1002/cpt.982 29243223 PMC5887840

[26] Antonia SJ Balmanoukian A Brahmer J Ou SI Hellmann MD Kim SW. Clinical activity, tolerability, and long-term follow-up of durvalumab in patients with advanced NSCLC. J Thorac Oncol. (2019) 14:1794–806. 10.1016/j.jtho.2019.06.010 31228626

[27] Feng Y Masson E Dai D Parker SM Berman D Roy A. Model-based clinical pharmacology profiling of ipilimumab in patients with advanced melanoma. Br J Clin Pharmacol. (2014) 78:106–17. 10.1111/bcp.12323 24433434 PMC4168385

[28] Weber JS O'Day S Urba W Powderly J Nichol G Yellin M. Phase I/II study of ipilimumab for patients with metastatic melanoma. J Clin Oncol. (2008) 26:5950–6. 10.1200/JCO.2008.16.1927 19018089

[29] Longoria TC Tewari KS. Evaluation of the pharmacokinetics and metabolism of pembrolizumab in the treatment of melanoma. Expert Opin Drug Metab Toxicol. (2016) 12:1247–53. 10.1080/17425255.2016.1216976 27485741 PMC5613934

[30] Agrawal S Feng Y Roy A Kollia G Lestini B. Nivolumab dose selection: challenges, opportunities, and lessons learned for cancer immunotherapy. J Immunother Cancer. (2016) 4:72. 10.1186/s40425-016-0177-2 27879974 PMC5109842

[31] Brahmer JR Drake CG Wollner I Powderly JD Picus J Sharfman WH. Phase I study of single-agent anti-programmed death-1 (MDX-1106) in refractory solid tumors: safety, clinical activity, pharmacodynamics, and immunologic correlates. J Clin Oncol. (2010) 28:3167–75. 10.1200/JCO.2009.26.7609 20516446 PMC4834717

[32] Herbst RS Soria JC Kowanetz M Fine GD Hamid O Gordon MS. Predictive correlates of response to the anti-PD-L1 antibody MPDL3280A in cancer patients. Nature. (2014) 515:563–7. 10.1038/nature14011 25428504 PMC4836193

[33] Powles T Eder JP Fine GD Braiteh FS Loriot Y Cruz C. MPDL3280A (anti-PD-L1) treatment leads to clinical activity in metastatic bladder cancer. Nature. (2014) 515:558–62. 10.1038/nature13904 25428503

[34] Mizugaki H Yamamoto N Murakami H Kenmotsu H Fujiwara Y Ishida Y. Phase I dose-finding study of monotherapy with atezolizumab, an engineered immunoglobulin monoclonal antibody targeting PD-L1, in Japanese patients with advanced solid tumors. Invest New Drugs. (2016) 34:596–603. 10.1007/s10637-016-0371-6 27363843 PMC5007272

[35] Lutzky J Antonia SJ Blake-Haskins A Li X Robbins PB Shalabi AM A phase 1 study of MEDI4736, an anti–PD-L1 antibody, in patients with advanced solid tumors. J Clin Oncol. (2014) 32:3001 10.1200/jco.2014.32.15_suppl.3001

[36] Song X Pak M Chavez C Liang M Lu H Schwickart M Pharmacokinetics and pharmacodynamics of MEDI4736, a fully human anti-programmed death ligand 1 (PD-L1) monoclonal antibody, in patients with advanced solid tumors. J Clin Oncol. (2015) 33:e14009 10.1200/jco.2015.33.15_suppl.e14009

[37] Bensalem A Ternant D. Pharmacokinetic variability of therapeutic antibodies in humans: a comprehensive review of population pharmacokinetic modeling publications. Clin Pharmacokinet. (2020). 10.1007/s40262-020-00874-2. [Epub ahead of print]. 32170579

[38] Hurkmans DP Basak EA van Dijk T Mercieca D Schreurs MWJ Wijkhuijs AJM. A prospective cohort study on the pharmacokinetics of nivolumab in metastatic non-small cell lung cancer, melanoma, and renal cell cancer patients. J Immunother Cancer. (2019) 7:192. 10.1093/annonc/mdz253.099 31324223 PMC6642527

[39] Vugt Mv Greef Rd Freshwater T Mangin E Aarle Fv Kondic A Immunogenicity of pembrolizumab (pembro) in patients (pts) with advanced melanoma (MEL) and non-small cell lung cancer (NSCLC): pooled results from KEYNOTE-001, 002, 006, and 010. J Clin Oncol. (2016) 34:3063 10.1200/JCO.2016.34.15_suppl.3063

[40] Agrawal S Statkevich P Bajaj G Feng Y Saeger S Desai DD. Evaluation of immunogenicity of nivolumab monotherapy and its clinical relevance in patients with metastatic solid tumors. J Clin Pharmacol. (2017) 57:394–400. 10.1002/jcph.818 27557786

[41] Kverneland AH Enevold C Donia M Bastholt L Svane IM Nielsen CH. Development of anti-drug antibodies is associated with shortened survival in patients with metastatic melanoma treated with ipilimumab. Oncoimmunology. (2018) 7:e1424674. 10.1080/2162402X.2018.1424674 29721387 PMC5927482

[42] Chatterjee MS Elassaiss-Schaap J Lindauer A Turner DC Sostelly A Freshwater T. Population pharmacokinetic/pharmacodynamic modeling of tumor size dynamics in pembrolizumab-treated advanced melanoma. CPT Pharmacometrics Syst Pharmacol. (2017) 6:29–39. 10.1002/psp4.12140 27896938 PMC5270297

[43] Herbst RS Baas P Kim DW Felip E Perez-Gracia JL Han JY. Pembrolizumab versus docetaxel for previously treated, PD-L1-positive, advanced non-small-cell lung cancer (KEYNOTE-010): a randomised controlled trial. Lancet. (2016) 387:1540–50. 10.1016/S0140-6736(15)01281-7 26712084

[44] Joseph RW Gangadhar TC Puzanov I Robert C Hamid O Dummer R Model-based analysis of the relationship between pembrolizumab (MK-3475) exposure and efficacy in patients with advanced or metastatic melanoma. J Clin Oncol. (2015) 33:3068 10.1200/jco.2015.33.15_suppl.3068

[45] Robert C Ribas A Wolchok JD Hodi FS Hamid O Kefford R. Anti-programmed-death-receptor-1 treatment with pembrolizumab in ipilimumab-refractory advanced melanoma: a randomised dose-comparison cohort of a phase 1 trial. Lancet. (2014) 384:1109–17. 10.1016/S0140-6736(14)60958-2 25034862

[46] Ribas A Puzanov I Dummer R Schadendorf D Hamid O Robert C. Pembrolizumab versus investigator-choice chemotherapy for ipilimumab-refractory melanoma (KEYNOTE-002): a randomised, controlled, phase 2 trial. Lancet Oncol. (2015) 16:908–18. 10.1016/S1470-2045(15)00083-2 26115796 PMC9004487

[47] Turner DC Kondic AG Anderson KM Robinson AG Garon EB Riess JW. Pembrolizumab exposure-response assessments challenged by association of cancer cachexia and catabolic clearance. Clin Cancer Res. (2018) 24:5841–9. 10.1158/1078-0432.CCR-18-0415 29891725

[48] Li H Yu J Liu C Liu J Subramaniam S Zhao H. Time dependent pharmacokinetics of pembrolizumab in patients with solid tumor and its correlation with best overall response. J Pharmacokinet Pharmacodyn. (2017) 44:403–14. 10.1007/s10928-017-9528-y 28573468

[49] Feng Y Wang X Bajaj G Agrawal S Bello A Lestini B. Nivolumab exposure-response analyses of efficacy and safety in previously treated squamous or nonsquamous non-small cell lung cancer. Clin Cancer Res. (2017) 23:5394–405. 10.1158/1078-0432.CCR-16-2842 28916617

[50] Bellesoeur A Ollier E Allard M Hirsch L Boudou-Rouquette P Arrondeau J. Is there an exposure-response relationship for nivolumab in real-world NSCLC patients? Cancers (Basel). (2019) 11:1784. 10.3390/cancers11111784 31766292 PMC6895963

[51] Bajaj G Gupta M Feng Y Statkevich P Roy A. Exposure-response analysis of nivolumab in patients with previously treated or untreated advanced melanoma. J Clin Pharmacol. (2017) 57:1527–33. 10.1002/jcph.962 28800166 PMC5697692

[52] Wang X Feng Y Bajaj G Gupta M Agrawal S Yang A. Quantitative characterization of the exposure-response relationship for cancer immunotherapy: a case study of nivolumab in patients with advanced melanoma. CPT Pharmacometrics Syst Pharmacol. (2017) 6:40–8. 10.1002/psp4.12133 28019090 PMC5270290

[53] Motzer RJ Rini BI McDermott DF Redman BG Kuzel TM Harrison MR. Nivolumab for metastatic renal cell carcinoma: results of a randomized phase II trial. J Clin Oncol. (2015) 33:1430–7. 10.1200/JCO.2014.59.0703 25452452 PMC4806782

[54] Basak EA Koolen SLW Hurkmans DP Schreurs MWJ Bins S Oomen-de Hoop E. Correlation between nivolumab exposure and treatment outcomes in non-small-cell lung cancer. Eur J Cancer. (2019) 109:12–20. 10.1016/j.ejca.2018.12.008 30654225

[55] Feng Y Roy A Masson E Chen TT Humphrey R Weber JS. Exposure-response relationships of the efficacy and safety of ipilimumab in patients with advanced melanoma. Clin Cancer Res. (2013) 19:3977–86. 10.1158/1078-0432.CCR-12-3243 23741070

[56] Wolchok JD Neyns B Linette G Negrier S Lutzky J Thomas L. Ipilimumab monotherapy in patients with pretreated advanced melanoma: a randomised, double-blind, multicentre, phase 2, dose-ranging study. Lancet Oncol. (2010) 11:155–64. 10.1016/S1470-2045(09)70334-1 20004617

[57] Ascierto PA Del Vecchio M Robert C Mackiewicz A Chiarion-Sileni V Arance A. Ipilimumab 10 mg/kg versus ipilimumab 3 mg/kg in patients with unresectable or metastatic melanoma: a randomised, double-blind, multicentre, phase 3 trial. Lancet Oncol. (2017) 18:611–22. 10.1016/S1470-2045(17)30231-0 28359784

[58] Bi Y Liu J Furmanski B Zhao H Yu J Osgood C. Model-informed drug development approach supporting approval of the 4-week (Q4W) dosing schedule for nivolumab (Opdivo) across multiple indications: a regulatory perspective. Ann Oncol. (2019) 30:644–51. 10.1093/annonc/mdz037 30715147

[59] Food and Drug Administration Guidance for Industry: Exposure–Response Relationships—Study Design, Data Analysis, and Regulatory Applications. Available online at: https://www.fda.gov/regulatory-information/search-fda-guidance-documents/exposure-response-relationships-study-design-data-analysis-and-regulatory-applications (accessed May 15, 2020).

[60] Freshwater T Kondic A Ahamadi M Li CH de Greef R de Alwis D. Evaluation of dosing strategy for pembrolizumab for oncology indications. J Immunother Cancer. (2017) 5:43. 10.1186/s40425-017-0242-5 28515943 PMC5433037

[61] Zhao X Suryawanshi S Hruska M Feng Y Wang X Shen J. Assessment of nivolumab benefit-risk profile of a 240-mg flat dose relative to a 3-mg/kg dosing regimen in patients with advanced tumors. Ann Oncol. (2017) 28:2002–8. 10.1093/annonc/mdx235 28520840 PMC5834087

[62] Deng R Bumbaca D Pastuskovas CV Boswell CA West D Cowan KJ. Preclinical pharmacokinetics, pharmacodynamics, tissue distribution, and tumor penetration of anti-PD-L1 monoclonal antibody, an immune checkpoint inhibitor. MAbs. (2016) 8:593–603. 10.1080/19420862.2015.1136043 26918260 PMC4966836

[63] Goldstein DA Ratain MJ. Alternative dosing regimens for atezolizumab: right dose, wrong frequency. Cancer Chemother Pharmacol. (2019) 84:1153–5. 10.1007/s00280-019-03971-7 31630223

[64] Zhao X Ivaturi V Gopalakrishnan M Shen J Feng Y Statkevich P Abstract CT101: A model-based exposure-response (E-R) assessment of a nivolumab (NIVO) 4-weekly (Q4W) dosing schedule across multiple tumor types. Cancer Res. (2017) 77(Suppl. 13):CT101 10.1158/1538-7445.AM2017-CT101

[65] Lala M Li TR de Alwis DP Sinha V Mayawala K Yamamoto N. A six-weekly dosing schedule for pembrolizumab in patients with cancer based on evaluation using modelling and simulation. Eur J Cancer. (2020) 131:68–75. 10.1016/j.ejca.2020.02.016 32305010

[66] Garon EB Reinmuth N Falchero L Garcia YG Hureaux J Gore I CheckMate 384: Phase IIIb/IV trial of nivolumab (nivo) 480 mg Q4W versus 240 mg Q2W after ≤ 12 months of nivo in previously treated advanced NSCLC. J Clin Oncol. (2019) 37:100 10.1200/JCO.2019.37.8_suppl.100

[67] Lebbe C Meyer N Mortier L Marquez-Rodas I Robert C Rutkowski P. Evaluation of two dosing regimens for nivolumab in combination with ipilimumab in patients with advanced melanoma: results from the phase IIIb/IV CheckMate 511 trial. J Clin Oncol. (2019) 37:867–75. 10.1200/JCO.18.01998 30811280 PMC6455714

[68] Reck M Ciuleanu T-E Dols MC Schenker M Zurawski B Menezes J Nivolumab (NIVO) + ipilimumab (IPI) + 2 cycles of platinum-doublet chemotherapy (chemo) vs 4 cycles chemo as first-line (1L) treatment (tx) for stage IV/recurrent non-small cell lung cancer (NSCLC): CheckMate 9LA. J Clin Oncol. (2020) 38:9501 10.1200/JCO.2020.38.15_suppl.9501

[69] Lala M Akala O Chartash E Kalabis M Su S-C Alwis DPd editors. CT042 - Pembrolizumab 400 mg Q6W dosing: First clinical outcomes data from Keynote-555 cohort B in metastatic melanoma patients. AACR Annual Meeting (2020). (2020). April 28, 20202020.

[70] Paz-Ares L Dvorkin M Chen Y Reinmuth N Hotta K Trukhin D. Durvalumab plus platinum-etoposide versus platinum-etoposide in first-line treatment of extensive-stage small-cell lung cancer (CASPIAN): a randomised, controlled, open-label, phase 3 trial. Lancet. (2019) 394:1929–39. 31590988 10.1016/S0140-6736(19)32222-6

[71] Hellmann MD Paz-Ares L Bernabe Caro R Zurawski B Kim SW Carcereny Costa E. Nivolumab plus ipilimumab in advanced non-small-cell lung cancer. N Engl J Med. (2019) 381:2020–31. 10.1056/NEJMoa1910231 31562796

[72] Ratain MJ Goldstein DA. Time is money: optimizing the scheduling of nivolumab. J Clin Oncol. (2018) 2018:JCO1800045. 10.1200/JCO.18.00045 30148658

[73] Renner A Burotto M Rojas C. Immune checkpoint inhibitor dosing: can we go lower without compromising clinical efficacy? J Glob Oncol. (2019) 5:1–5. 10.1200/JGO.19.00142 31348737 PMC6690659

[74] Dudnik E Moskovitz M Agbarya A Kuznetsov T Shochat T Urban D OA11.06 Alternative nivolumab (N) duration and scheduling in advanced non-small cell lung cancer (aNSCLC): real-life data. J Thoracic Oncol. (2019) 14:S235–6. 10.1016/j.jtho.2019.08.469

[75] Yoo SH Keam B Kim M Kim SH Kim YJ Kim TM. Low-dose nivolumab can be effective in non-small cell lung cancer: alternative option for financial toxicity. ESMO Open. (2018) 3:e000332. 10.1136/esmoopen-2018-000332 30094065 PMC6069908

[76] Sehgal K Bulumulle A Brody H Gill R Macherla S Qilleri A Association of extended dosing intervals or delays in pembrolizumab-based regimens with survival outcomes in advanced non-small cell lung cancer. Clin Lung Cancer. (2020). 10.1101/2020.03.31.20048637. [Epub ahead of print]. PMC7273162 32653295

[77] Remon J Lopes G Camps C. How sustainable are new treatment strategies for NSCLC? Lancet Respir Med. (2019) 7:733–5. 10.1016/S2213-2600(19)30184-5 31208953

[78] Workman P Draetta GF Schellens JHM Bernards R. How much longer will we put up with $100,000 cancer drugs? Cell. (2017) 168:579–83. 10.1016/j.cell.2017.01.034 28187281

[79] Ogungbenro K Patel A Duncombe R Nuttall R Clark J Lorigan P. Dose rationalization of pembrolizumab and nivolumab using pharmacokinetic modeling and simulation and cost analysis. Clin Pharmacol Ther. (2018) 103:582–90. 10.1002/cpt.875 28913853

[80] Goldstein DA Gordon N Davidescu M Leshno M Steuer CE Patel N. A phamacoeconomic analysis of personalized dosing vs fixed dosing of pembrolizumab in firstline PD-L1-positive non-small cell lung cancer. J Natl Cancer Inst. (2017) 109. 10.1093/jnci/djx063 29059432

[81] Bayle A Besse B Annereau M Bonastre J. Switch to anti-programmed cell death protein 1 (anti-PD-1) fixed-dose regimen: what is the economic impact? Eur J Cancer. (2019) 113:28–31. 10.1016/j.ejca.2019.02.016 30965212

[82] Hall E Zhang J Kim EJ Hwang G Chu G Bhatia S. Economics of alternative dosing strategies for pembrolizumab and nivolumab at a single academic cancer center. Cancer Med. (2020) 9:2106–12. 10.1002/cam4.2888 31994335 PMC7064089

[83] Peer CJ Goldstein DA Ratain MJ Figg WD A modeling and simulation study of less frequent dosing of nivolumab 480 mg. J Clin Oncol. (2019) 37:3115 10.1200/JCO.2019.37.15_suppl.3115

